# Improving the Identification of the Preclinical Stages of Spinocerebellar Ataxia Type 2

**DOI:** 10.3390/tomography12070092

**Published:** 2026-06-24

**Authors:** Camilo Mora-Batista, Cruz Vargas-De-León, Ramón Reyes-Carreto, Frank J. Carrillo-Rodes, José Alberto Álvarez-Cuesta

**Affiliations:** 1 Facultad de Matemáticas, Universidad Autónoma de Guerrero, Chilpancingo de los Bravo 39087, Mexico; 22251526@uagro.mx; 2 División de Investigación, Hospital Juárez de México, Ciudad de México 07760, Mexico; 3 Laboratorio de Modelación Bioestadística para la Salud, Sección de Estudios de Posgrado e Investigación, Escuela Superior de Medicina, Instituto Politécnico Nacional, Ciudad de México 11340, Mexico; 4 Centro de Investigación y Rehabilitación de las Ataxias Hereditarias, VPWP+RM5, Holguín 80100, Cuba; frankjrodes@gmail.com (F.J.C.-R.); cuesta140560@gmail.com (J.A.Á.-C.)

**Keywords:** spinocerebellar ataxia, multinomial logistic regression, magnetic resonance image, synthetic minority over-sampling technique, neuroimaging biomarkers

## Abstract

Background: Spinocerebellar ataxia type 2 (SCA2) is an inherited neurodegenerative disorder characterized by progressive cerebellar degeneration. One difficulty in treating this disease lies in identifying preclinical carriers: individuals who carry the pathogenic ATXN2 mutation but remain asymptomatic with respect to motor manifestations. Though magnetic resonance imaging (MRI) has proven valuable in supporting the diagnosis of ataxia, traditional univariate approaches using linear measurements have shown limited ability to capture the complex anatomical changes that occur across the disease spectrum, particularly during the preclinical phase. Methods: This study employed a comprehensive multivariate approach to improve the classification of individuals across the SCA2 spectrum. We developed a multinomial logistic regression model incorporating multiple linear measurements derived from magnetic resonance imaging to discriminate between healthy controls (n = 72), preclinical carriers (n = 17), and patients with manifest SCA2 (n = 61). To mitigate inherent class imbalance, particularly in the smaller preclinical subgroup, we implemented the Synthetic Minority Over-sampling Technique (SMOTE), generating a balanced dataset that enhances the model’s ability to discern the distinctive anatomical features. This was compared to the model applied to the unbalanced data. An improvement was observed when applying SMOTE. Results: The multivariate model demonstrated discriminatory performance, achieving an overall accuracy of 80.7%. The ability to identify healthy controls (AUC: 0.96), preclinical individuals (AUC: 0.75), and clinical individuals (AUC: 95%). This represents an advance over previous univariate approaches, which have had difficulty capturing the neurodegenerative changes characteristic of the preclinical stage. Conclusions: By integrating multiple neuroimaging biomarkers into a multivariable model, this study provides a tool for early identification of preclinical SCA2 carriers. The ability to accurately classify these individuals opens an opportunity for early therapeutic intervention before irreversible neurological deterioration occurs. This approach shows promise for optimizing clinical trial design and personalized care in SCA2.

## Simple Summary

Spinocerebellar ataxia type 2 (SCA2) is a hereditary neurodegenerative disease that progressively affects coordination and balance. Detecting individuals who carry the mutation before symptoms appear is essential for early intervention and future therapeutic strategies. In this study, we used multiple magnetic resonance imaging measurements combined with a multivariable statistical model to classify healthy individuals, preclinical carriers, and patients with manifest SCA2. To improve the identification of the smaller preclinical group, we applied the Synthetic Minority Over-sampling Technique (SMOTE), which enhanced classification performance. The proposed approach achieved high accuracy and showed better performance than previous methods based on single imaging measurements. These findings suggest that combining neuroimaging biomarkers with machine learning strategies may support earlier detection and improve clinical management of SCA2.

## 1. Introduction

Hereditary ataxias constitute a clinically and genetically diverse group of disorders. The autosomal dominant forms, known as spinocerebellar ataxias (SCAs), are characterized by progressive degeneration of the cerebellum and brainstem. These are classified numerically according to the discovery of their genetic loci, with around 50 variants identified, including the most common subtypes, such as SCA1, SCA2, SCA3, SCA6, SCA7, and DRPLA [1,2,3,4,5,6].

Globally, spinocerebellar ataxia type 2 (SCA2) is the second most prevalent autosomal dominant ataxia. Its highest concentration is reported in Cuba, where a founder effect has been identified, particularly in the province of Holguín. In this province, 578 patients from 106 families represent 86.78% of all cases of hereditary ataxia in the country, and 95.7% of families are carriers of the SCA2 mutation [3]. The disease is caused by an abnormal expansion of the CAG trinucleotide (32 to 77 repeats) in the ATXN2 gene on chromosome 12. The resulting ataxin-2 protein accumulates mainly in Purkinje neurons, leading to severe neuropathological changes characterized by early olivopontocerebellar atrophy and degeneration in the substantia nigra, thalamus, and spinal cord [7,8,9,10].

Clinical onset typically occurs in the third or fourth decade of life. Crucially, SCA2 mutation carriers experience a prolonged preclinical period. During this asymptomatic stage, neurodegenerative processes are already underway, manifesting as a spectrum of structural changes. While some carriers show no detectable atrophy, others exhibit very mild cerebral atrophy, but none have yet developed the clinical symptoms that define Stage 1 on the Scale for the Assessment and Rating of Ataxia (SARA). When symptoms do appear, they primarily reflect cerebellar dysfunction, including gait ataxia, dysarthria, and dysmetria [11,12,13,14,15,16,17,18]. The disease course is aggressive, typically spanning 10–15 years, although specialized rehabilitation can mitigate its progression.

Standardized clinical assessment relies on scales like SARA and the Inventory of Non-Ataxia Symptoms (INAS) to quantify deficits and track progression [19,20]. However, a major limitation of these clinical scales is their inability to detect the preclinical stage, where neurodegeneration, as evidenced by imaging, is already occurring. This gap underscores the urgent need for objective tools to detect presymptomatic manifestations [21,22,23,24,25,26,27,28,29].

The gold standard for diagnosis remains genetic testing to confirm the CAG expansion. While essential for definitive diagnosis, this test has significant limitations: it provides only binary (presence/absence) information, cannot monitor disease progression, and is often inaccessible for large-scale family screening due to cost. Consequently, an estimated 50% of hereditary ataxia prevalence may go undocumented [30].

Neuroimaging has become indispensable as it can visualize the characteristic cerebellar and brainstem atrophy. Notably, MRI can detect these structural changes even in the presymptomatic stage, offering a window into the early neurodegenerative process that genetic testing cannot provide. MRI’s superior soft-tissue contrast makes it the modality of choice over CT for evaluating the posterior fossa [31,32,33,34,35,36,37,38,39,40,41,42,43,44,45]. A broad consensus in the literature confirms that structural atrophy in SCAs is well-documented and linked to clinical symptoms.

Advanced MRI techniques are being explored as objective biomarkers for neurodegeneration, with the potential to serve as surrogate endpoints in clinical trials for novel therapies. The development of such therapies is critical, as no cure currently exists. However, the significant heterogeneity across SCA subtypes necessitates individualized treatment approaches, and the validation of these therapies requires more precise assessment tools than current clinical scales [29].

While quantitative volumetric MRI has consistently shown differences between patients and controls, a critical gap remains: the lack of an objectively validated, clinically applicable diagnostic threshold. This gap is partly due to methodological constraints in existing research, such as small sample sizes, heterogeneous groups, variable acquisition protocols, and a reliance on high-field (3T+) MRI systems not always available in routine practice [18]. Our previous work established a binary threshold using the anteroposterior diameter of the midbrain (DAPMES) to distinguish healthy controls from manifest SCA2 patients. However, this dichotomous approach is insufficient to characterize the full pathophysiological continuum, particularly the crucial preclinical stage with its inherent heterogeneity [46].

To address these limitations, the present study proposes a more robust and clinically rigorous approach. We expand our previous binary model to a multinomial model that includes three groups: healthy controls, preclinical carriers, and patients with manifest SCA2. This model will be based on a set of three accessible linear measurements: the anteroposterior diameter of the midbrain (DAPMES), the anteroposterior diameter of the pons (DAPP), the vertical diameter of the vermis (DHIV), and the individual’s age. To tackle the common challenge of small sample sizes in the preclinical group, which leads to bias, we will implement the Synthetic Minority Oversampling Technique (SMOTE) to generate a synthetically balanced cohort for multinomial logistic regression analysis. The model’s performance was quantitatively validated by comparing the balanced cohort with the unbalanced baseline using the Brier score, which ensured that oversampling did not lead to overfitting, while also yielding a better calibration curve. This approach, based on simple linear measurements, has the potential to be applied in conventional clinical settings, thus improving its translational value.

In summary, this study employs a simple, reproducible, and objective manual linear measurement method to develop a multinomial model that specifically targets the pressing need to identify and characterize preclinical carriers of SCA2. This improved methodology will allow us to: differentiate between the three groups based on multivariate anatomical profiles; improve the prediction of symptom onset; and establish more objective outcome measures for future therapeutic trials.

## 2. Materials and Methods

### 2.1. Patients and Methods

This observational, analytical case–control study was conducted from February 2021 to June 2022 at two participating institutions in Holguín province, Cuba: the “Lucía Íñiguez Landín” Clinical-Surgical Hospital and the “Carlos Juan Finlay” Center for Research and Rehabilitation of Ataxias (CIRAH). The study population comprised SCA2 gene carriers from Holguín province, derived from a national epidemiological study that identified 484 cases in this region. All provincial gene carriers were invited to participate and were categorized into three groups: a Preclinical Group (mutation carriers with a SARA score ≤ 2.5; this cohort encompasses the pre-manifest spectrum, including prodromal cases with subtle signs that do not yet meet the criteria for manifest ataxia), an Ataxic Group (carriers with florid ataxia symptoms and SARA score ≥ 3), and a Control Group matched by age strata within a 2-year range.

Of the total patients carrying the ATXN2 gene mutation, 21.7% (n = 17) are in the preclinical stage. This subgroup presents a fundamental conceptual and clinical dilemma: they are asymptomatic carriers of the genetic mutation, which implies that, from a molecular perspective, they are affected by the pathological expansion of the CAG trinucleotide in the ATXN2 gene; however, from a clinical and functional perspective, they have not yet manifested the disease, as they do not present the motor symptoms that define stage 1 on the SARA scale [47]. This borderline condition, in which neurodegeneration may already be in progress, with or without measurable atrophy, but the symptoms remain unexpressed, is precisely the group of greatest interest for the present study. The accurate identification and classification of these preclinical individuals is crucial, as it opens a window of opportunity to implement early therapeutic strategies, such as those developed in the CIRAH rehabilitation program. These interventions allow patients to prepare for the course of the disease and take measures to mitigate its progression, thus improving their quality of life before neurological deterioration becomes established.

**Figure 1:**
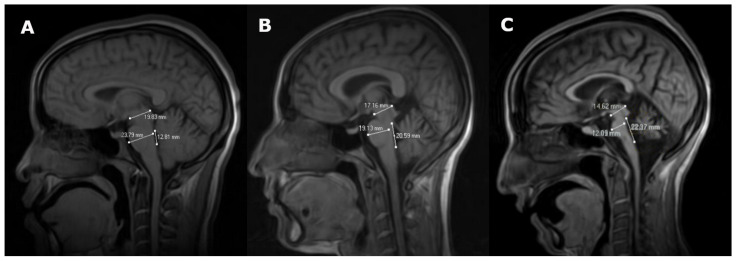
This shows how the DAPMES, DAPP, and DHIV diameters are obtained via MRI. (**A**) shows measurements for a control subject, (**B**) for a preclinical SCA2 patient, and (**C**) for a clinical SCA2 patient. The progression of atrophy is evident from (**A**–**C**).

The subtle progression of structural neurodegeneration at this preclinical stage, which often goes unrecognized by the SARA scale, is illustrated in Figure 1 These radiological images demonstrate a clear atrophic gradient from the control stage (A) to the ataxic stage (C), highlighting specific biomarkers such as DAPMES, DAPP, and DHIV, which are discussed in greater detail in subsequent sections and provide a more sensitive indicator of disease progression than clinical scores alone.

#### 2.1.1. Screening of the Control Group and Patients

Control subjects were selected through optimal stratified matching, preferentially chosen from close relatives with negative genetic test results from family screening. When no suitable family member was available or consented to participate, the first patient undergoing MRI who met inclusion criteria was selected as control. This matching approach accounted for age’s direct relationship with olivopontocerebellar degeneration degrees without gender restrictions.

All participants underwent comprehensive neurological evaluation by two CIRAH neurologists specialized in ataxia diagnosis and follow-up. The assessment included detailed personal and family medical history, symptom onset date, disease duration, and clinical evaluation using the SARA. Control group subjects received identical clinical evaluations to exclude signs and symptoms of neurodegenerative diseases.

Both groups underwent brain Magnetic Resonance Imaging at the “Lucía Íñiguez Landín” Clinical-Surgical Hospital using identical image acquisition protocols throughout the study period. The multidisciplinary research team included specialists from various fields who contributed to different aspects of the investigation.

For the patients, their medical history, family medical history, date of onset of symptoms, duration of the disease, and clinical evaluation of the disease using the SARA scale were assessed.

#### 2.1.2. Conducting the Cranial MRI Study

For the MRI study of the brain performed on all participants, a standardized imaging acquisition protocol was established for this research purpose using a Philips device, model Panorama 0.23 T. The protocol included axial, sagittal, and coronal T1-weighted sequences, FFE 3D-24/90, with a slice thickness of 5.5 mm following established anatomical reference lines.

Data acquisition was performed using a 256 × 256 matrix and a field of view (FOV) of 230 mm to ensure adequate spatial resolution for manual morphometry. The potential measurement error associated with the low-field system was mitigated by using a standardized anatomical reference protocol, which ensured high intra-observer consistency in the recorded linear diameters. Further details of the protocol can be found in the doctoral thesis [48].

#### 2.1.3. Manual Measurement of Neuroimaging Biomarkers

All measurements were performed on digital midsagittal T1-weighted MRI images to ensure maximum measurement accuracy. The imaging analysis software Imagis version 1.13 was used for all linear measurements.

Three linear measurements MRI-derived neuroimaging biomarkers were assessed as follows:

1. Anteroposterior Diameter of the Midbrain (DAPMES): Measured in millimeters (mm) on the midsagittal slice. A line representing the vertical axis of the midbrain was drawn, followed by a perpendicular line extending from the anterior midbrain surface to the midpoint between the superior and inferior colliculi.
2. Anteroposterior Diameter of the Pons (DAPP): Measured in mm on the midsagittal slice. A line representing the vertical axis of the pons was drawn, followed by a perpendicular line from the most convex anterior portion to the most convex posterior portion, without reaching the base of the fourth ventricle triangle.
3. Vertical Diameter of the Vermis (DHIV): The maximum craniocaudal diameter of the cerebellar vermis, measured in mm on the midsagittal slice.

The data collection process was supervised by F.J.C.R. and J.A.A.C. All measurements were performed independently by three experienced neuroradiologists (each with >15 years in neuroimaging), who were blinded to participants’ age, sex, and clinical status, acted as independent evaluators, and were not part of the core research team. In case of discrepancy, the final value was the average of both measurements. More details on the measurements can be found in the doctoral thesis [48].

Inter-rater reliability or ICC (Intraclass Correlation Coefficient) among the three clinical experts was evaluated using a two-way mixed-effects model under the consistency definition. Both single-measure [ICC(3,1)] and mean-measure [ICC(3,k)] reliability were assessed in the final sample of 150 participants. Individual assessments demonstrated excellent agreement, with ICC(3,1) values of 0.9391 (95% CI: 0.9200–0.9500) for DAPMES, 0.9818 (95% CI: 0.9800–0.9900) for DAPP, and 0.9790 (95% CI: 0.9700–0.9800) for DHIV (all *p* < 0.001). When applying the multi-expert consensus protocol, mean-measure reliability increased further, reaching near-perfect levels with ICC(3,k) values of 0.9789 (95% CI: 0.9700–0.9800) for DAPMES, 0.9939 (95% CI: 0.9900–1.0000) for DAPP, and 0.9929 (95% CI: 0.9900–0.9929) for DHIV. In accordance with [49] guidelines, these results indicate excellent inter-rater reliability, confirming that the consensus-averaged dataset provides a highly stable, consistent, and robust foundation for subsequent multinomial classification analyses.

### 2.2. Exploratory Data Analysis and Variance Inflation Factor

**Figure 2:**
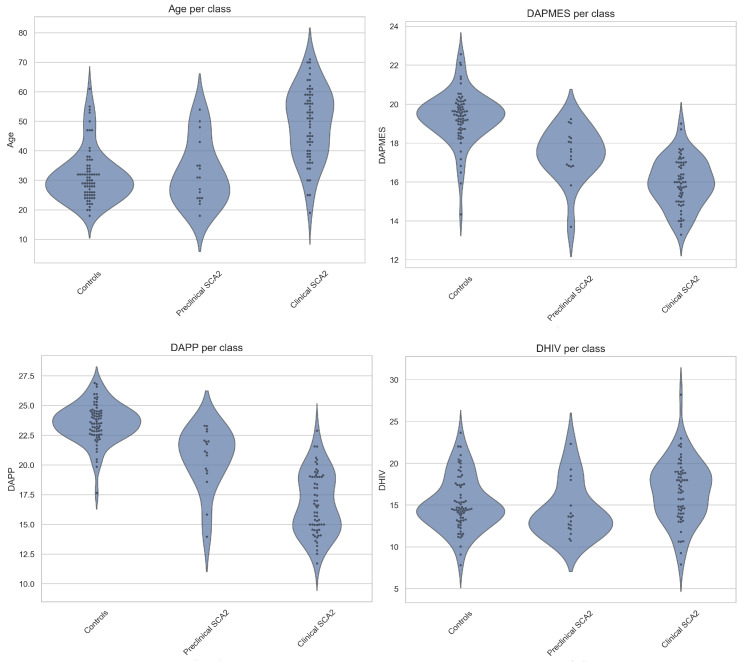
Comparative analysis of age and morphometric variables across clinical categories. The distribution of Age, DAPMES, DAPP, and DHIV is shown for each group: Healthy Controls, Preclinical SCA2, and Clinical SCA2. While Preclinical and Clinical stages represent the progression of Spinocerebellar Ataxia Type 2, Healthy Controls serve as the baseline. The combination of violin and swarm plots highlights the density and individual variability within each clinical classification.

**Figure 3:**
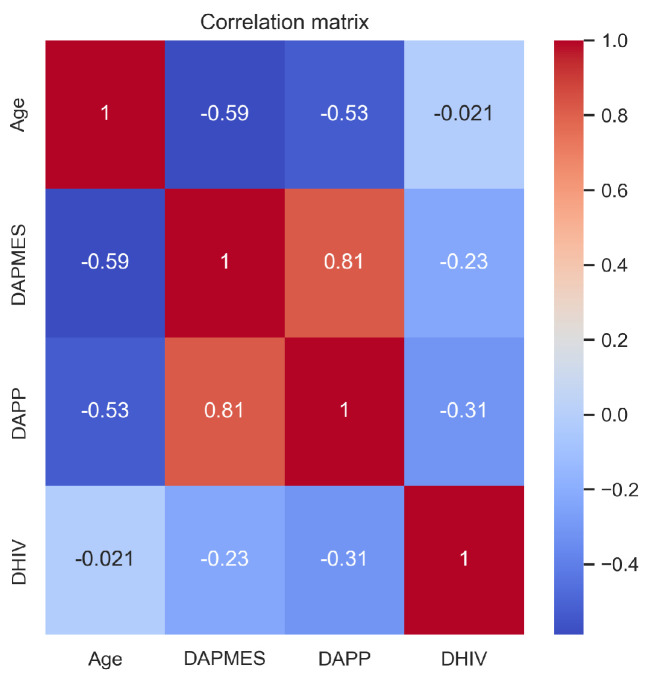
Pearson correlation matrix of age and MRI biomarkers. The heatmap displays the correlation coefficients between Age and the morphometric variables (DAPMES, DAPP, and DHIV). Red indicates a strong positive correlation, while blue represents a strong negative correlation, as shown in the color scale. High collinearity is observed between DAPMES and DAPP (r=0.81), whereas Age shows a moderate inverse relationship with both measures.

**Figure 4:**
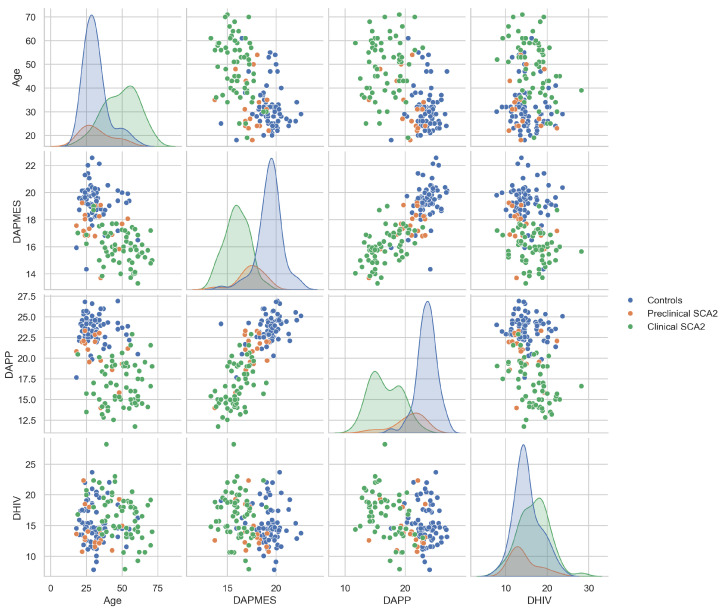
Pairwise relationships and distribution density of age and MRI biomarkers by clinical category. The diagonal panels display Kernel Density Estimate (KDE) plots, showing the distribution of each variable (Age, DAPMES, DAPP, and DHIV) stratified by clinical group: Healthy Controls (blue), Preclinical SCA2 (orange), and Clinical SCA2 (green). The off-diagonal scatter plots illustrate the bivariate relationships between variables, highlighting the clustering patterns and linear trends across the different clinical classifications. This visualization provides a comprehensive overview of how morphometric changes align with the clinical categorization of the study participants.

To evaluate the discriminatory power of the selected MRI biomarkers and their relationship with age, a comprehensive exploratory data analysis was conducted. This phase aims to characterize the morphological transition from healthy states to disease manifestation, providing a visual and statistical basis for subsequent predictive modeling. By combining distribution analyses (Figure 2), correlation matrices (Figure 3), and multivariate pairwise relationships (Figure 4), we examined the structural patterns defining each clinical category. Furthermore, to guarantee the statistical robustness of the multinomial regression and eliminate redundancies among highly correlated brainstem measures, a multicollinearity analysis was performed using the variance inflation factor (VIF).

Figure 2 shows the following: The age distribution indicates that the control group and the preclinical SCA2 group share similar demographic profiles, with a high concentration around age 30. In contrast, the clinical SCA2 group shows a notable shift toward older ages (mean close to 55 years) and greater dispersion, reflecting the progressive nature of the disease, where motor symptoms typically manifest in later stages of life. A clear and progressive reduction in DAPMES is observed as the disease spectrum progresses. While controls maintain robust values (median of 20 mm), the preclinical group already shows early signs of atrophy, and the clinical group exhibits the most significant volume reduction. This linear tendency suggests that DAPMES is a sensitive early biomarker of brainstem neurodegeneration in SCA2. The diameter of the pons (DAPP) shows a significant separation between the groups. There is a clear transition from controls to the preclinical stage, culminating in pronounced atrophy in clinical patients (median < 15 mm). The minimal overlap between the control group and the clinical group highlights the diagnostic utility of this measure for confirming the pons degeneration characteristic of the disease. Contrary to brainstem diameters, the height of the fourth ventricle (DHIV) shows a tendency toward widening or expansion. Clinical patients present significantly higher values, indicating ventricular dilatation secondary to atrophy of the cerebellar tissue and the surrounding brainstem. The preclinical group shows an intermediate distribution, which appears to correspond to a state of morphological transition.

The scatter plots in Figure 4 suggest strong linear correlations between the structural biomarkers (DAPMES and DAPP), indicating coordinated atrophy in the brainstem. The diagonal densities confirm that, although age is a confounding factor, morphological biomarkers provide a clear separation between healthy and clinical subjects, while preclinical subjects consistently occupy the transition space, justifying the use of multivariate models to improve their detection. To examine this correlation between DAPMES and DAPP, we obtain the correlation matrix shown in Figure 3.

#### Multicollinearity Analysis

Analysis of the correlation matrix revealed significant multicollinearity between DAPMES and DAPP (r = 0.81). This relationship suggests redundancy in the data that could destabilize the multinomial regression coefficients and increase standard errors. To formally address this observation, the Variance Inflation Factor (VIF) was calculated. This step ensures transparency in feature selection, allowing for the identification and, if necessary, exclusion of variables with high VIF values (e.g., >5) to ensure the robustness and interpretability of the clinical model.

Analysis of the Variance Inflation Factor (VIF) confirmed the presence of no critical multicollinearity in the model, with all clinical predictors falling below the conservative threshold of 5. The values obtained were 1.63 for Age, 3.32 for DAPMES, 3.20 for DAPP, and 1.17 for DHIV. Although the correlation matrix indicated a relationship between DAPMES and DAPP, these results demonstrate that the redundancy is not sufficient to destabilize the regression coefficients, guaranteeing the robustness of the predictors and the interpretability of each variable independently.

### 2.3. Statistical Analysis

Data are presented as mean (standard deviation, SD) for continuous variables and as frequency (percentage) for categorical variables. Inter-subgroup comparisons of demographic and clinical characteristics were performed using one-way ANOVA or the Kruskal–Wallis test for non-normally distributed continuous variables in Python version 3.13.9. Categorical variables were compared using the chi-square test, with post hoc pairwise comparisons adjusted using the Bonferroni correction.

Due to marked class imbalance, particularly in the preclinical group (n = 17), the Synthetic Minority Over-sampling Technique (SMOTE) was applied exclusively to the training dataset to generate synthetic samples for the minority classes, resulting in a balanced training set while preserving the original test set distribution.

A multinomial logistic regression model was fitted to the SMOTE-balanced training set to classify participants into three categories (healthy control, preclinical SCA2, and clinical SCA2) using the three linear neuroimaging biomarkers DAPMES, DAPP, DHIV, and patient age as predictors. The reference category was set as healthy controls. We emphasize that we used cross-validation to evaluate and validate the performance of the multiple regression model. The same procedure was performed on the data without applying SMOTE to compare the improvement achieved by balancing the samples.

We looked at several key metrics, precision, accuracy, recall, and F1 score, calculating these for each individual class, as well as macro and weighted averages. We also examined the confusion matrix and, for each class, plotted the One-vs-Rest ROC curves along with their AUC values. To check whether the predicted probabilities were reliable, we calculated the Brier score and compared the calibration curves.

Odds ratios with 95% confidence intervals and corresponding *p*-values were calculated for each predictor; only the best model is reported here. All analyses were performed using Python 3 (libraries: pandas for data handling, imbalanced-learn for SMOTE, statsmodels for statistical *p*-value inference, and scikit-learn for modeling and evaluation). A two-sided *p*-value < 0.05 was considered statistically significant.

## 3. Results

### 3.1. Study Population and Demographic Characteristics

The study cohort comprised a total of 150 participants, including 72 healthy controls, 61 symptomatic SCA2 patients, and 17 preclinical SCA2 individuals (Table 1). The distribution by sex was comparable between the different groups, with a general predominance of female participants (87 women and 63 men) and no statistically significant differences in the sex ratios between the diagnostic categories.

The mean age differed significantly between the groups. Healthy controls had a lower mean age than patients with symptomatic SCA2, and preclinical individuals had intermediate values. Specifically, patients with symptomatic SCA2 were older than controls, consistent with disease progression, while preclinical individuals were younger and asymptomatic at the time of evaluation.

Regarding clinical severity, symptomatic SCA2 patients were classified according to the SARA into mild, moderate, and severe stages, reflecting a wide spectrum of disease involvement. As expected, preclinical individuals were considered presymptomatic and assigned a SARA score of 0 for analytical purposes.

Linear neuroimaging biomarkers derived from magnetic resonance imaging showed distinctive patterns between groups. For example, the measurements DAPMES and DAPP follow an order relationship in their average; see Table 1. Patients with symptomatic SCA2 had reduced measurements of the brainstem and cerebellum compared to healthy controls, and preclinical individuals showed intermediate morphometric values, suggesting early structural involvement before obvious clinical manifestation.

### 3.2. Derivation of the Multivariate Model for Classifying the Status of SCA2

To extend the previously proposed univariate risk score for atrophy [46], a multivariate modeling approach was adopted to characterize the probability of belonging to different clinical stages of SCA2. Given the presence of three clinically relevant groups (healthy controls = Controls, preclinical SCA2 = Preclinical SCA2, and symptomatic SCA2 = Clinical SCA2), a multinomial logistic regression model was used. This approach allows for the simultaneous estimation of relative risk profiles at different stages of the disease, based on morphometric measurements of the brainstem and demographic variables.

A multinomial logistic regression model was fitted to estimate the probability of belonging to different clinical stages of SCA2 patients (preclinical and clinical), using healthy controls as the reference category. The model in Table 2 demonstrated excellent goodness of fit overall, with a pseudo-$R^{2}$ of 0.6484 and a highly significant likelihood ratio test compared to the null model (Log-Likelihood Ratio, LLR *p*-value = $2.455\times 10^{-36}$), indicating acceptable explainability of the included predictors.

In both comparisons, the anteroposterior diameter of the midbrain (DAPMES) and the anteroposterior diameter of the pons (DAPP) were found to be the most robust predictors. Both variables showed high negative coefficients and high statistical significance (*p* < 0.001), indicating that reductions in brainstem dimensions were strongly associated with a higher probability of belonging to preclinical or clinical SCA2 groups compared to controls.

Age showed a differential effect between disease stages, attaining statistical significance only in the comparison between clinical SCA2 and controls, suggesting a stage-dependent influence.

In terms of the horizontal diameter of the vermis, a differential pattern was observed between the stages of the disease. When comparing preclinical individuals with controls, the DHIV showed a positive but not significant coefficient (0.2056; 95% CI: −0.046 to 0.457; *p* = 0.109), indicating that this measure does not adequately discriminate between the two groups in the early stages. However, when comparing clinical patients with controls, DHIV showed a positive and statistically significant association (0.3222; 95% CI: 0.042 to 0.603; *p* < 0.042), suggesting that vermis atrophy consolidates and becomes detectable only when the disease has progressed to the symptomatic phase. This finding reinforces the idea that brainstem structures, particularly DAPMES and DAPP, are more sensitive biomarkers for early detection, while the vermis may more accurately reflect progression to the clinical phase. Taken together, these results highlight the importance of a multimodal approach combining brainstem and cerebellar measurements to capture the full continuum of SCA2 neurodegeneration.

**Figure 5:**
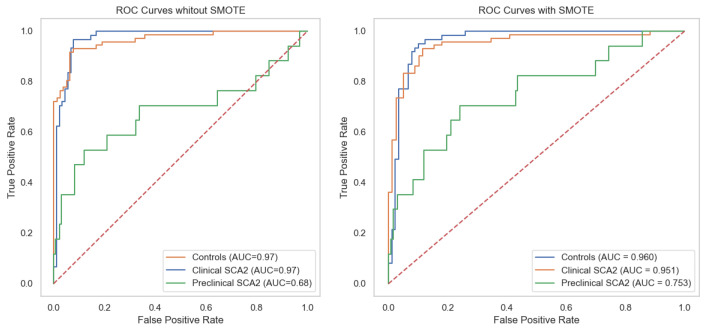
One-vs-Rest Receiver Operating Characteristic (ROC) curves for the multinomial logistic regression model. The red dashed line is the non-discrimination line. The plots compare model performance without SMOTE and with SMOTE for distinguishing healthy controls, preclinical SCA2, and clinical SCA2 patients.

Figure 5 shows the ROC curves of One-vs-Rest of the multinomial model for each clinical category, on the left side without SMOTE and on the right side with SMOTE. The implementation of SMOTE yielded a substantial improvement in the model’s discriminative capacity, particularly for the minority class. While the original model showed an AUC of 0.68 for the Preclinical SCA2 group, the use of synthetic oversampling increased this value to 0.75, enabling better capture of biological signals in early stages. Conversely, the Controls and Clinical SCA2 categories maintained exceptional and stable performance, with AUC values transitioning from 0.97 to 0.96 and 0.95, respectively. These results demonstrate that SMOTE balances the model’s learning process, reducing bias toward majority classes and strengthening the detection of the preclinical stage. This high discriminatory performance underscores that the selected morphometric biomarkers, such as the linear measurements of the midbrain and pons, accurately capture progressive structural atrophy. These results demonstrate that such variables provide a robust separation between disease stages, validating their clinical utility for identifying even subtle changes during the preclinical phase.

### 3.3. Brier Score and Calibrations Curve of Both Models with and Without SMOTE

The initial evaluation of the multinomial logistic regression model without SMOTE yielded a Brier Score of 0.2410 (95% CI: 0.1782–0.3085). In a three-class classification context, a score near 0.25 indicates that the model’s probabilistic predictions are only slightly better than random chance. The corresponding calibration curve reveals significant reliability issues, particularly for the Preclinical SCA2 group, where the model consistently underestimated disease probability due to the limited sample size (n=17). This baseline performance underscores the negative impact of class imbalance on the model’s ability to provide calibrated clinical risks.

Implementation of the SMOTE resulted in a substantial improvement in model calibration. The cross-validated Brier Score decreased to 0.0901 (95% CI: 0.0443–0.1358). This marked reduction in the Brier Score signifies a superior alignment between the predicted probabilities and the actual observed frequencies. By balancing the training distribution, SMOTE allowed the logistic regression algorithm to better estimate the decision boundaries, shifting the model from a state of high uncertainty to one of high probabilistic reliability.

**Figure 6:**
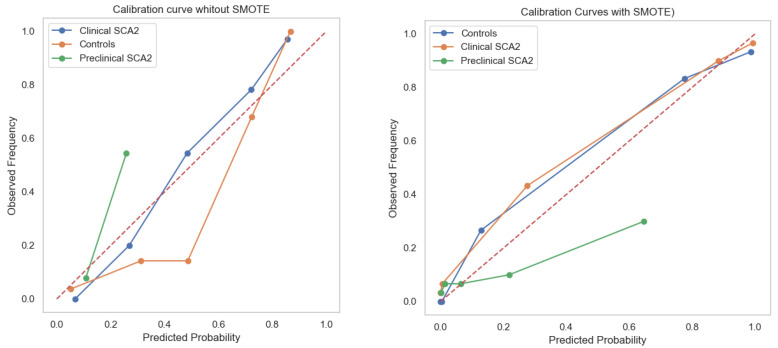
Reliability diagrams comparing model calibration before and after SMOTE. Calibration curves for healthy controls, preclinical SCA2, and clinical SCA2. The red dashed line is the perfect calibration line. Synthetic oversampling (**right**) effectively reduces the deviations observed in the original unbalanced model (**left**), improving overall diagnostic reliability.

The visual comparison of the calibration plots in Figure 6 highlights the corrective effect of oversampling. In the model with SMOTE, the curves for Healthy Controls and Clinical SCA2 show an excellent fit to the ideal $45^{\circ }$ diagonal across the entire probability spectrum. Although the Preclinical SCA2 group still exhibits some deviation, its trajectory is considerably more stable compared to the original model. SMOTE effectively mitigated the “pessimistic” bias toward the preclinical class, expanding the range of predicted probabilities and providing a more nuanced risk assessment for patients in early stages of neurodegeneration.

From a clinical perspective, the transition from a Brier Score of 0.24 to 0.09 is critical for the utility of morphometric biomarkers. While the ROC curves (discrimination) proved that variables like DAPMES and DAPP can separate the groups, the improved calibration ensures that the specific probability assigned to a patient is a trustworthy reflection of their actual clinical status. These results demonstrate that combining structural brain measurements with adequate sampling techniques like SMOTE provides a robust and transparent framework for the objective classification of SCA2 progression.

### 3.4. Validation

The multinomial logistic regression model was evaluated using a cross-validation framework across the full dataset (n=150), achieving an overall accuracy of 80.67% (95% CI: 73.61% to 86.19%). Table 3 shows the confusion matrix. Performance metrics by class revealed a high discriminative capacity for the established stages of the disease. Healthy controls were identified with a sensitivity of 86.89% and a high specificity of 93.26%, resulting in a robust F1-score of 0.88. Similarly, symptomatic SCA2 patients exhibited stable performance, with a sensitivity of 83.33% and a Positive Predictive Value (PPV) of 89.55% (F1-score = 0.86), confirming the model’s reliability in identifying clinically manifest disease. In contrast, the preclinical group representing 11.33% of the sample posed a greater classification challenge, yielding a sensitivity of 47.06% and a PPV of 33.33%. Despite this lower sensitivity, the high specificity (87.97%) and Negative Predictive Value (92.86%) for this group suggest that the model is effective at ruling out the preclinical stage in healthy or symptomatic individuals. The weighted F1-score of 0.818 reflects a strong overall performance, while the macro F1-score of 0.712 accounts for the inherent heterogeneity and the expected difficulty in detecting subtle morphometric changes during the early preclinical phase.

Together, the ROC analysis, confusion matrix, and class-specific performance metrics provide a consistent assessment of the model’s ability to distinguish between the clinical stages of SCA2 following the implementation of SMOTE. The ROC curves showed high AUC values across all categories, with the preclinical group achieving an AUC of 0.75, indicating better separability. The confusion matrix revealed that most misclassifications occurred between adjacent disease stages specifically between preclinical and control individuals, while high accuracy was maintained for the symptomatic group. Notably, the model achieved a negative predictive value of 92.86% for the preclinical stage, supporting its clinical plausibility. Overall, the balance between sensitivity and specificity suggests a substantial level of agreement between the predicted and actual clinical status, consistent with a screening oriented tool designed to prioritize individuals for specialized follow up rather than provide a definitive diagnosis.

The optimal probability thresholds were below 0.5, which is expected in a multinomial setting where class probabilities are distributed across multiple categories and influenced by class balancing techniques such as SMOTE. For class Control, a probability threshold of 0.232 indicates that observations with predicted probabilities above this value are more likely to belong to this class under the optimal sensitivity specificity trade off. Similarly, thresholds of 0.266 and 0.222 were identified for classes Preclinical and Clinical, respectively, reflecting the probability levels at which each class is best discriminated in a one-vs-rest setting. These values are consistent with a multinomial framework, where probabilities are distributed across multiple classes.

## 4. Discussion

This study represents an advance in the neuroimaging of spinocerebellar ataxia type 2 (SCA2) by developing a multinomial logistic regression model that integrates three simple linear MRI biomarkers (DAPMES, DAPP, and DHIV) to discriminate between controls, preclinical, and clinical cases. This multivariate approach provides a practical tool for early disease staging, in contrast to previously reported binary models [46], which are limited to discriminating between healthy controls and symptomatic patients.

Classifying the preclinical condition in SCA2 is especially critical because neurodegeneration begins years before clinical motor symptoms, creating a potential therapeutic window for interventions that modify the disease. Detecting carriers at risk with good sensitivity allows for longitudinal monitoring, assessment of progression, and, eventually, early initiation of treatments with greater neuronal reserve. Previous studies have shown that MRI measures (such as brainstem volumes) and fluid biomarkers detect subtle changes in preclinical individuals, but the absence of accessible and validated methods limits their routine use in clinical practice, particularly in regions with high prevalence such as Cuba.

The scientific community has employed various approaches for early detection in SCA2, predominantly MRI volumetry, liquid biomarkers, and functional signals. Deep learning approaches for automatic brainstem segmentation have revealed severe atrophy in preclinical carriers (nearly 50% reduction in the pons compared to controls) [50]. Serum light neurofilament levels correlate with clinical severity and appear elevated near the expected age of onset in preclinical individuals, with sensitivities of approximately 88% at certain cut-off values [51]. Other studies have used deep learning with Monte Carlo dropout on electrooculogram signals to discriminate presymptomatic individuals from controls and patients, achieving accuracies of approximately 81% and emphasizing non-invasive methods based on sensors [52]. The sensitivity of MRI for early changes is confirmed by these developments and evidence of cerebellar and pontine atrophy in preclinical patients [53], though they frequently necessitate high-field MRI, standardized protocols, and complex analysis.

In contrast, our multinomial model, which is based on simple manual linear measurements, complements these advanced techniques and provides an acceptable alternative in constrained settings. It achieves a respectable AUC value (0.753) in preclinical studies without requiring expensive infrastructure. Our findings are in line with earlier research that linked the diameters of the brainstem (midbrain and pons) to the clinical severity of ataxias. They also support the value of these easily accessible linear measurements in creating population reference standards for midbrain diameters.

Although our multinomial model offers a useful tool for SCA2 staging, particularly as an initial step prior to confirmatory genetic testing, it is not offered as a conclusive diagnostic tool but rather as a useful tool in resource-constrained environments.

Since the model performs exceptionally well overall, its interpretation goes beyond performance metrics. The combination of DAPMES, DAPP, and DHIV effectively captures the early degenerative changes that mostly occur in the brainstem, according to the high sensitivity for identifying the preclinical stage. This pattern probably reflects the pontocerebellar pathways’ selective vulnerability in SCA2, where gliosis and axonal loss start years before motor symptoms manifest. However, given the known phenotypic heterogeneity of this disease, the slightly lower specificity in some cases raises the possibility that individual anatomical variations or comorbidities could cause overlap between controls and preclinical carriers.

Some unexpected results, such as the misclassification of certain preclinical individuals into the control or clinical categories, are particularly interesting. These inconsistent cases may not simply be model noise, but could reflect real differences in the rate of progression or in the neural reserve of each carrier. Carriers with greater cognitive reserve or smaller CAG repeats may show less linear atrophy despite being close to the age of onset, suggesting that our model captures not only the current state but also aspects related to penetrance and individual progression.

Compared to previous studies that report greater accuracy with volumetric or artificial intelligence methods, our approach does not aim to exceed them in accuracy, but rather to offer a low-cost, easy-to-implement complementary tool [45,53]. While deep learning-based models require technological infrastructure that is not available in many centers, especially in endemic regions such as Cuba, our model confirms the validity of simple linear measurements and positions them as a useful first filter [26]. This represents a clear practical advantage, although at the cost of a minor loss of detail in detecting very small changes.

From a clinical perspective, this model could have a significant impact on real-world practice. First, it would serve as a screening tool for relatives of diagnosed patients, allowing for the prioritization of individuals for confirmatory genetic testing and the optimization of resources. Additionally, it would facilitate the longitudinal monitoring of preclinical carriers in ataxia clinics, helping to determine the optimal time to initiate potential disease-modifying therapies once they become available. Errors in the model, primarily confusion between preclinical and control cases, would in practice necessitate additional testing (genetic or higher-resolution), which is clinically acceptable and preferable to missing cases within the therapeutic range.

Despite these contributions, our study has inherent limitations. The small size of the preclinical sample (n = 17) may influence the accuracy of this important category and increase the variability of estimates. The transverse design prevents the evaluation of individual progression or model stability over time. Manual linear measurements entail possible interobserver variability, although this was controlled for using standardized protocols. The future longitudinal validations, larger cohorts, and semiautomatic improvements could improve its clinical applicability and clinical trials.

The cross-sectional nature of the study and the small size of the preclinical sample limit the generalizability of the findings to other populations with SCA2. As the disease exhibits significant genetic and phenotypic variability even within families with the same CAG expansion, our results may not fully reflect the heterogeneity observed in more diverse cohorts or in regions with different genetic profiles. Additionally, the lack of correlation with biomarkers in body fluids (such as light neurofilament) or detailed clinical scales prevents an assessment of the extent to which linear measurements capture the true neurodegenerative workload. These limitations emphasize that, while the model offers practical value in resource-limited environments, it should be interpreted with care and validated prospectively before being considered a routine tool in clinical practice.

## 5. Conclusions

In conclusion, this study presents a practical and accessible model based on simple linear MRI measurements that effectively discriminates between clinical stages of SCA2. The implementation of SMOTE proved to be a decisive factor in the model’s success, significantly reducing the Brier Score from 0.24 to 0.09 and enhancing the detection of the preclinical stage. The final model achieved an overall accuracy of 80.67%, supported by high discriminative performance with Area Under the Curve (AUC) values of 0.96 for healthy controls, 0.95 for symptomatic patients, and 0.75 for preclinical carriers. This approach offers a valuable screening tool for early disease identification, particularly in resource-limited environments. By providing a reliable probability of disease status, especially for early-stage detection, the model enables timely interventions such as genetic counseling and rehabilitation programs before major neurological deterioration occurs. While further longitudinal validation is encouraged, these results demonstrate that the combination of morphometric biomarkers and advanced sampling techniques provides a robust basis for integrating objective classification into the standard clinical practice for SCA2 management.

## Figures and Tables

**Table 1:** Demographic and clinical characteristics of the study population.

Variable	Controls	Preclinical SCA2	Clinical SCA2	*p*-Value
	**n = 72**	**n = 17**	**n = 61**	
Sex (Male) ^1^	33 (45.8%)	3 (17.6%)	22 (45.8%)	–
Age (years) ^2^	31.46 (8.86)	32.29 (10.72)	36.74 (14.94)	<0.001
SARA score ^1^	NA	NA	NA	NA
Preclinical stage	NA	17 (21.7%)	NA	NA
Mild stage	NA	NA	40 (51.2%)	NA
Moderate stage	NA	NA	19 (24.3%)	NA
Severe stage	NA	NA	2 (2.5%)	NA
Disease duration (years) ^2^	NA	NA	10.16 (6.52)	NA
DAPMES (mm) ^2^	19.36 (1.30)	17.45 (1.34)	16.14 (1.10)	<0.001
DAPP (mm) ^2^	23.56 (1.61)	20.59 (2.59)	17.06 (2.69)	<0.001
DHIV (mm) ^2^	15.17 (3.15)	14.36 (3.25)	16.73 (3.91)	<0.016

^1^ Absolute frequency (n) and percentage (%). ^2^ Mean (standard deviation). NA (not applicable). Percentages for preclinical and clinical stages are relative to the total number of genetic mutation carriers (n=78).

**Table 2:** Multinomial logistic regression results for SCA2 clinical status with Odds Ratios.

Predictor	Coef.	OR	SE	*p*-Value	95% CI (OR)
*Preclinical SCA2 vs. Controls (y = 1)*					
Age	0.1176	1.125	0.036	0.001	[1.047, 1.208]
DAPMES	0.0425	1.043	0.345	0.902	[0.531, 2.050]
DAPP	−0.3747	0.687	0.182	0.039	[0.481, 0.981]
DHIV	0.2056	1.228	0.128	0.109	[0.955, 1.579]
*Clinical SCA2 vs. Controls (y = 2)*					
Age	0.0525	1.054	0.038	0.163	[0.979, 1.134]
DAPMES	1.0049	2.732	0.330	0.002	[1.430, 5.217]
DAPP	0.7147	2.044	0.231	0.002	[1.301, 3.212]
DHIV	0.3222	1.380	0.143	0.024	[1.043, 1.828]

Coefficients are reported relative to the study group, on the left side without SMOTE and on the right side with SMOTE. OR: Odds Ratio; CI: confidence interval; SE: standard error. Note: Constant terms omitted for brevity.

**Table 3:** Confusion matrix of the multinomial classification model for SCA2 clinical status with SMOTE.

Actual Class	Control	Preclinical SCA2	Clinical SCA2
Control	60	10	2
Preclinical SCA2	5	8	4
Clinical SCA2	2	6	53

## Data Availability

Due to the sensitive nature of the clinical and MRI data analyzed in this study, the datasets are not publicly available. Data sharing is restricted by the institutional review board to protect participant confidentiality.

## Abbreviations

The following abbreviations are used in this manuscript:

AUC	Area Under the Curve
DAPMES	Anteroposterior Diameter of the Midbrain
DAPP	Anteroposterior Diameter of the Pons
DHIV	Vertical Diameter of the Vermis
INAS	Inventory of Non-Ataxia Symptoms
ICC	intraclass correlation coefficient
MRI	Magnetic Resonance Imaging
ROC	Receiver Operating Characteristic
SARA	Scale for the Assessment and Rating of Ataxia
SCA2	Spinocerebellar Ataxia Type 2
SECIHTI	Secretaría de Ciencia, Humanidades, Tecnología e Innovación
FOV	field of view
LLR	Log-Likelihood Ratio
